# Public health risks from subclinical variant CJD

**DOI:** 10.1371/journal.ppat.1006642

**Published:** 2017-11-30

**Authors:** Abigail B. Diack, Robert G. Will, Jean C. Manson

**Affiliations:** 1 The Roslin Institute & R(D)SVS, University of Edinburgh, Easter Bush, United Kingdom; 2 National CJD Research and Surveillance Unit, University of Edinburgh, Edinburgh, United Kingdom; 3 The Centre for Dementia Prevention, University of Edinburgh, Edinburgh, United Kingdom; 4 Edinburgh Neuroscience, University of Edinburgh, Edinburgh, United Kingdom; Washington University School of Medicine, UNITED STATES

## Introduction

Variant Creutzfeldt-Jakob disease (vCJD) is a zoonotic prion disease thought to have been transmitted to humans through the consumption of food products contaminated with bovine spongiform encephalopathy (BSE) in the 1980s and/or early 1990s. As with all prion disorders, it is a fatal neurodegenerative disease arising from conversion of the normal cellular form of the prion protein PrP^C^, encoded by the prion gene (*PRNP*), to an abnormal form associated with disease (PrP^Sc^). Prion diseases have long asymptomatic incubation periods ranging from years to decades. Extensive studies involving both natural and experimental animal diseases have demonstrated that infectivity is present during the subclinical phase and may be transmitted between individuals. While the cases of clinical vCJD currently appear to be in decline, one of the current challenges is defining the prevalence of subclinical disease and the risk this poses to both individuals and the general population.

## 1. vCJD: Current perspective

In 1996, the first cases of vCJD were identified through systematic surveillance in the United Kingdom in collaboration with other European countries. vCJD typically occurs in young adults, and susceptibility is associated with the codon 129 polymorphism of *PRNP* (methionine [M]/valine [V]), with 177 out of 178 clinical cases being 129MM [1]. However, experimental transmission studies indicate that all genotypes may be susceptible and that 129MV and 129VV individuals may have long asymptomatic incubation periods [2]. Although the annual number of deaths due to vCJD peaked in 2000 with 28 deaths, this has since decreased, with only two deaths since 2011; one in 2013, and one in 2016 [1]. However, the latest case, in 2016, is the first instance of probable and/or definite clinical vCJD in a 129MV individual, raising concerns that a second wave of vCJD may occur in individuals of this genotype [1].

In addition to primary cases, thought to be directly acquired from BSE, three clinical cases of vCJD have been identified in 129MM individuals who had received nonleucoreduced red cell concentrates from UK donors who were asymptomatic at the time of donation but later died of vCJD. In addition, evidence of subclinical infection has been found in two other individuals, both of whom had a 129MV genotype [3]. One of these asymptomatic individuals showed no evidence of PrP^Sc^ in the brain, but evidence of PrP^Sc^ was identified in the spleen and a cervical lymph node [4]. Bioassay of the spleen material in wild-type and transgenic mice expressing human PrP (129MM) confirmed that the spleen carried vCJD infectivity (Table 1) [5]. Additional studies that have utilised protein misfolding cyclic amplification (PMCA; a protein amplification technique analogous to PCR that measures prion seeding activity) have also provided evidence of widespread prion seeding activity in a range of tissues in the asymptomatic 129MV individual (Table 1) [6].

**Table 1:** Tissues identified as containing abnormal PrP and/or infectivity in clinical and subclinical vCJD patients.

Tissue	Clinical vCJD patients (129MM)	Subclinical vCJD patient (129MM)	Subclinical vCJD patient (129MV)
Frontal cortex	+^1, 2, 3^	+^1,3^	-^2^
Pituitary gland	+^1^	ND	+^2^
Cervical lymph node	+^1, 2^	ND	+^1, 2^
Tonsil	+^1, 2, 3^	ND	+^2^
Appendix	+^1, 2^	+^1^	+^2^
Distal ileum	+^1, 2^	ND	+^2^
Spleen	+^1, 2, 3^	ND	+^1, 2, 3^
Thymus	+^1^	ND	+^2^
Lung	+^2^	ND	+^2^
Heart	+^2^	ND	-^2^
Liver	+^1, 2^	ND	+^2^
Kidney	+^1, 2^	ND	-^2^
Salivary gland	+^2^	ND	+^2^
Pancreas	+^1, 2^	ND	-^2^
Thyroid	+^2^	ND	-^2^
Adrenal gland	+^1, 2^	ND	-^2^
Bone marrow	+^2^	ND	-^2^
Skeletal muscle	+^1, 2^	ND	-^2^
Optic nerve	+^1^	ND	ND
Testis	+^2^	ND	ND
Ovary	+^1, 2^	ND	ND
Rectum	+^1^	ND	ND
Retina	+^1^	ND	ND
Skin	+^1^	ND	ND
Uterus	+^1^	ND	ND
Blood (plasma)	+^2^	+^2^	ND

Abnormal PrP and/or infectivity measured by immunohistochemistry/biochemical analysis,^1^ PMCA,^2^ or infectivity^3^ studies. Data compiled from [4–7,18–20]. +, positive; -, negative.

Abbreviations: M, methionine; ND, no data; PMCA, protein misfolding cyclic amplification; PrP, prion protein; V, valine; vCJD, variant Creutzfeldt-Jakob disease.

## 2. Abnormal PrP and transmission potential

Understanding the association between the presence of PrP^Sc^ and infectivity is essential in order to estimate disease prevalence and manage transmission risks. Retrospective appendix studies have indicated that 1 in 2,000 individuals have evidence of abnormal PrP in the appendix. The first studies of paraffin-embedded appendixes from preclinical vCJD patients demonstrated that abnormal PrP can be detected at least two years before clinical symptoms become apparent [7]. The first retrospective study reported a prevalence of approximately 1 in 4,000 individuals with abnormal PrP in their appendixes; of the three positive specimens identified, two were 129VV (Table 2) [8,9]. Two larger studies were subsequently performed. Appendix II and III both found a prevalence of approximately 1 in 2,000. Appendix II identified 16 positive samples that included a higher proportion of 129VV when compared with the normal population distribution and confirmed clinical cases of vCJD (Table 2) [10]. Appendix III was conducted on specimens that had been removed either prior to the BSE crisis or from individuals who were born in 1996 or later, at a time when food safety measures had been fully implemented. In this study, seven specimens containing abnormal PrP deposits were identified (Table 2) [11], and this raises questions about the interpretation of the abnormal PrP in relation to vCJD infection, particularly in those cohorts with presumably limited exposure to BSE. It is uncertain whether the positive immunohistochemical staining observed is vCJD specific, although such staining has not been observed in sporadic Creutzfeldt-Jakob disease (sCJD) [12]. One interpretation is that the period of human exposure to BSE was more extended than previously thought. These considerations are important for public health because individuals subclinically infected with vCJD may have the potential to transmit disease to others [8–10].

**Table 2:** Summary of the appendix survey results.

Study	No. of positive samples	Birth cohort	Appendix removal period	Codon 129 genotype	Sex
Appendix I [8,9]	3/12,674	-	1995–2000	VV (2)	-
Appendix II [10]	16/21,441	1941–1945	2000–2012	MM	M
		1946–1950		MM	M
		1951–1955		MM	M
		1956–1960		MV VV (2)	M F (2)
		1966–1970		VV (2) MV	F (2) M
		1971–1975		MV MV	M F
		1976–1980		MM (3)	M (3)
		1981–1985		MM MM	M F
Appendix III [11]	2/5,865	-	1977–1979	-	-
	5/10,074	1996–2000	2000–2014	-	-

-, no data.

Abbreviations: MM, methionine homozygous; MV, methionine/valine; VV, valine homozygous.

## 3. Subclinical prion disease

Subclinical disease is a well-studied phenomenon in prion diseases. Animal models demonstrate that during a long asymptomatic period, the level of infectivity increases in peripheral and central nervous system (CNS) tissue well before the onset of clinical disease [13]. During this period, infected tissues pose a risk of onward transmission, for example in humans via tissue transplant, contaminated surgical instruments, or infected blood.

A number of animal studies have investigated blood as a route of transmission of prion disease [3]. Seminal studies in sheep models of prion disease demonstrated that BSE could be transmitted via blood transfusion [14]. Further studies demonstrated that all blood components were capable of transmitting disease during the subclinical period [15]. The blood-transfusion–related human cases demonstrate that individuals of the 129MM genotype can transmit infectivity prior to developing clinical signs of disease.

Although the blood-transfusion–related cases of vCJD provide evidence of subclinical transmission, we still do not know at which point and in which tissues individuals first become infectious nor whether this will vary between codon 129 genotypes. Assessing the risk of iatrogenic transmission associated with subclinical vCJD is required to ascertain the potential for the transmission of disease between individuals.

## 4. Detecting subclinical disease

Although abnormal PrP can be detected by immunohistochemistry or seeding activity in a number of tissues from asymptomatic individuals, blood or urine are the only practicable substrates for the premortem identification of infected individuals [6]. A number of tests with varying specificity and sensitivity have been developed in order to detect vCJD infection by using blood or plasma from vCJD patients [16,17]. Recently, a test utilising a combination of a plasminogen bead capture system and PMCA that consistently detected vCJD prions from clinical vCJD cases with 100% sensitivity and 100% diagnostic specificity was developed [18]. This test also demonstrated an ability to detect prions in blood plasma from two subclinical blood donors (both 129MM); 1.3 and 2.6 years before clinical onset of disease. It is, however, unknown whether this test can detect prions in the blood or plasma of individuals with other codon 129 genotypes.

## 5. Determining risks to the individual and the population

The data from the Appendix studies indicate that up to 1 in 2,000 individuals in the UK may be subclinical carriers of vCJD. Whether subclinical vCJD infection leads to clinical disease is unknown and may depend on the recipient’s genotype, age, route, and/or level of infection. It is also unknown whether the clinical manifestation of the disease will vary in different genetic backgrounds and whether these individuals can transmit disease. The uncertainty surrounding these issues can only be addressed by continued surveillance, appropriate risk management, and the development of highly sensitive and specific markers of infection. To date, the only evidence of transmission of vCJD between individuals is via blood transfusion.

Continued human surveillance is required in order to identify new cases of vCJD, to promptly recognise any changes in clinical and/or pathological phenotype and to determine whether there are other routes of secondary transmission. At present, a number of risk management strategies are in place to reduce the risk of human-to-human transmission through blood. In the UK, these include leucoreduction of blood donations, donor deferral, and plasma sourcing from abroad. The development of blood tests could aid in allowing screening of blood donations or testing at-risk individuals. Concomitant with these approaches is a need to continue research into determining the nature of infectivity and the level of infectivity throughout the natural course of vCJD infection. It is essential that we do not become complacent and continue both surveillance and risk management strategies in both human and animal prion diseases.
